# Development and validation of a machine learning-based predictive model to improve the prediction of inguinal status of anal cancer patients: A preliminary report

**DOI:** 10.18632/oncotarget.10749

**Published:** 2016-07-21

**Authors:** Berardino De Bari, Mauro Vallati, Roberto Gatta, Laëtitia Lestrade, Stefania Manfrida, Christian Carrie, Vincenzo Valentini

**Affiliations:** ^1^ Radiation Oncology Department, Centre Hospitalier Universitaire Vaudois-CHUV, Lausanne, Switzerland; ^2^ University of Huddersfield, School of Computing and Engineering, Huddersfield, UK; ^3^ Radiation Oncology Department, Catholic University of Sacred Heart, Rome, Italy; ^4^ Service de Radiothérapie, Léon Bérard Cancer Center, Lyon, France; ^5^ Radiation Oncology Department, Hôpitaux universitaires de Genève-HUG, Geneva, Switzerland

**Keywords:** anal canal cancer, radiochemotherapy, prophylactic inguinal irradiation, machine learning, predicitive models

## Abstract

**Introduction:**

The role of prophylactic inguinal irradiation (PII) in the treatment of anal cancer patients is controversial. We developped an innovative algorithm based on the Machine Learning (ML) allowing the tailoring of the prescription of PII.

**Results:**

Once verified on the independent testing set, J48 showed the better performances, with specificity, sensitivity, and accuracy rates in predicting relapsing patients of 86.4%, 50.0% and 83.1% respectively (vs 36.5%, 90.4% and 80.25%, respectively, for LR).

**Methods:**

We classified 194 anal cancer patients with Logistic Regression (LR) and other 3 ML techniques based on decision trees (J48, Random Tree and Random Forest), using a large set of clinical and therapeutic variables. We tested obtained ML algorithms on an independent testing set of 65 anal cancer patients. TRIPOD (Transparent Reporting of a multivariable prediction model for Individual Prognosis or Diagnosis) methodology was used for the development, the Quality Assurance and the description of the experimental procedures.

**Conclusion:**

In an internationally approved quality assurance framework, ML seems promising in predicting the outcome of patients that would benefit or not of the PII. Once confirmed in larger and/or multi-centric databases, ML could support the physician in tailoring the treatment and in deciding if deliver or not the PII.

## INTRODUCTION

Anal canal carcinomas (ACCs) are rare, representing 2% of all digestive cancers and 6% of the ano-rectal cancers, but their incidence is increasing [1]. Since the publication of Randomized Controled Trials (RCTs), External Beam Radiotherapy (RT) +/- concomitant chemotherapy (CT) is the standard treatment for ACCs [2–4]. The role of the prophylactic inguinal irradiation (PII) is a controversial topic of debate: for N0 tumors, PII is considered effective, but it results in larger RT field sizes. Thus, it could contribute to higher risks of acute and late toxicity. A recent study by Ortholan et al., confirming the efficacy of the PII in preventing inguinal recurrences and its indication for all T3-4 tumors, concluded also that PII should be discussed for early-stage tumors, because they present a not-negligible 5-year inguinal recurrence rate of 12% when omitting PII, a rate that is substantially high considering the early stage of these cancers [5]. On the other hand, looking at the same figures, it could be easily argued that only 1/10 patients presenting an early stage ACC would really benefit of the PII, but all the patients receiving it are exposed to the potential risk of its acute and late toxicity.

Moreover, a study by Crowley et al. supports the use, for selected cases of ACC, of smaller than standard radiation fields, avoiding PII, in order to reduce acute and late toxicity [6], an attitude of particular interest in patients presenting an intrinsic higher risk of toxicity (*e.g.* HIV+ patients, elderly patients…) [7, 8]. Unfortunately, none of the available classical statistical techniques or predictive models allow the identification of patients presenting a higher risk of inguinal microscopic invasion (for example, higher than 5%).

Predictive models based on the Machine Learning (ML) and Artificial Intelligence (AI) techniques are being more and more adopted in the medical and bio-molecular field, as these methods have many attractive theoretic properties, specifically, the ability of analysing very large datasets and to detect non predefined relations such as nonlinear effects and/or interactions [9, 10]. Despite the growing interest of scientific community in exploring the potential of these techniques in the decision process in clinical oncology, any of these studies have never been addressed to the identification of a predictor of the patients that could more benefit of the PPI. This study was specifically addressed to the development and verification of a ML-based predictor to solve this clinical problem.

## RESULTS

### Participants (items 13a-13c, see Table 1 for the items of this and for the following sub-sessions)

**Table 1:** The TRIPOD checklist (adapted from [12])

Section/Topic	Item	Checklist Item
**Title and abstract**		
Title	1	Identify the study as developing and/or validating a multivariable prediction model, the target population, and the outcome to be predicted (D;V)*.
Abstract	2	Provide a summary of objectives, study design, setting, participants, sample size, predictors, outcome, statistical analysis, results, and conclusions (D;V).
**Introduction**		
Background and objectives	3a	Explain the medical context (including whether diagnostic or prognostic) and rationale for developing or validating the multivariable prediction model, including references to existing models (D;V).
	3b	Specify the objectives, including whether the study describes the development or validation of the model or both (D;V).
**Methods**		
Source of data	4a	Describe the study design or source of data (e.g., randomized trial, cohort, or registry data), separately for the development and validation data sets, if applicable (D;V).
	4b	Specify the key study dates, including start of accrual; end of accrual; and, if applicable, end of follow-up (D;V).
Participants	5a	Specify key elements of the study setting (e.g., primary care, secondary care, general population) including number and location of centres (D;V).
	5b	Describe eligibility criteria for participants (D;V).
	5c	Give details of treatments received, if relevant (D;V).
Outcome	6a	Clearly define the outcome that is predicted by the prediction model, including how and when assessed (D;V).
	6b	Report any actions to blind assessment of the outcome to be predicted (D;V).
Predictors	7a	Clearly define all predictors used in developing or validating the multivariable prediction model, including how and when they were measured (D;V).
	7b	Report any actions to blind assessment of predictors for the outcome and other predictors.
Sample size	8	Explain how the study size was arrived at (D;V).
Missing data	9	Describe how missing data were handled (e.g., complete-case analysis, single imputation, multiple imputation) with details of any imputation method (D;V).
Statistical analysis methods	10a	Describe how predictors were handled in the analyses (D).
	10b	Specify type of model, all model-building procedures (including any predictor selection), and method for internal validation (D).
	10c	For validation, describe how the predictions were calculated (V).
	10d	Specify all measures used to assess model performance and, if relevant, to compare multiple models (D;V).
	10e	Describe any model updating (e.g., recalibration) arising from the validation, if done (V).
Risk groups	11	Provide details on how risk groups were created, if done (D;V).
Development vs. validation	12	For validation, identify any differences from the development data in setting, eligibility criteria, outcome, and predictors (V).
**Results**		
Participants	13a	Describe the flow of participants through the study, including the number of participants with and without the outcome and, if applicable, a summary of the follow-up time. A diagram may be helpful (D;V).
	13b	Describe the characteristics of the participants (basic demographics, clinical features, available predictors), including the number of participants with missing data for predictors and outcome (D;V).
	13c	For validation, show a comparison with the development data of the distribution of important variables (demographics, predictors and outcome) (V).
Model development	14a	Specify the number of participants and outcome events in each analysis (D).
	14b	If done, report the unadjusted association between each candidate predictor and outcome (D).
Model specification	15a	Present the full prediction model to allow predictions for individuals (i.e., all regression coefficients, and model intercept or baseline survival at a given time point) (D).
	15b	Explain how to the use the prediction model (D).
Model performance	16	Report performance measures (with CIs) for the prediction model (D;V).
Model-updating	17	If done, report the results from any model updating (i.e., model specification, model performance) (V).
**Discussion**		
Limitations	18	Discuss any limitations of the study (such as non representative sample, few events per predictor, missing data) (D;V).
Interpretation	19a	For validation, discuss the results with reference to performance in the development data, and any other validation data (V).
	19b	Give an overall interpretation of the results, considering objectives, limitations, results from similar studies, and other relevant evidence (D;V).
Implications	20	Discuss the potential clinical use of the model and implications for future research (D;V).
**Other information**		
Supplementary information	21	Provide information about the availability of supplementary resources, such as study protocol, Web calculator, and data sets (D;V).
Funding	22	Give the source of funding and the role of the funders for the present study (D;V).

*Items relevant only to the development of a prediction model are denoted by “D”, items relating solely to a validation of a prediction model are denoted by “V”, and items relating to both are denoted “D;V”.

Nineteen patients received a Curative Inguinal Irradiation (CII), and 2/19 presented an inguinal relapse. Concerning the remaining 175 patients, 151 of them did not receive a PII and 24 received it. Finally, 13/151 patients (8.6%) and 3/24 pts (12.5%) presented an inguinal relapse. The 5-years inguinal-DFS in these 2 groups of patients rates were 87.5% and 90.7%, respectively (p=0.38).

Table 2 summarizes results in terms of specificity, sensitivity and accuracy of the 3 considered AI-based methods in identifying patients that would relapse.

**Table 2:** Performances of the 3 proposed machine learning techniques in identifying patients that would relapse (results on training set and on testing set are showed)

AI approaches (training set)*	False + (FP)	False - (FN)	True + (TP)	True - (TN)	Specificity %	Sensitivity%	Accuracy%
J48	39	41	29	121	75.6	41.4	65.2
Random Tree	31	4	66	129	80.6	94.3	84.8
Random Forest	16	5	65	144	90.0	92.9	90.9
**AI approaches** (independent testing set)**	**False + (FP)**	**False - (FN)**	**True + (TP)**	**True - (TN)**	**Specificity%**	**Sensitivity%**	**Accuracy%**
J48	8	3	3	51	86.4	50.0	83.1
Random Tree	12	5	1	47	79.7	16.7	73.9
Random Forest	9	4	2	50	84.8	33.3	80.0
LR	6	2	1	54	90.1	33.3	87.0
Always Negative	60	3	0	60	100.0	0.0	95.2

*The total number of patients in this table seems to be different from the total number of 194 pts only because of the Oversampling that has been applied. The real population accounted always for 194 pts.

** The total number of patients of the test set is 65.

Depending on the technique and the goal, the overall sensitivity, specificity, and accuracy rates of the ML techniques ranged between 41.3-94.3%, 75.6-90.0% and 65.2-90.9%, respectively, while the LR presented overall sensitivity, specificity, and accuracy rates of 36.5%, 94.8% and 80.2%, respectively.

The Random Forest was the best method in predicting patients that would relapse, with specificity, sensitivity, and accuracy rates of 90.0%, 92.9% and 90.9%, respectively (See Table 2).

Once verified on the independent testing set of 65 patients, the overall specificity, sensitivity, and accuracy rates of the ML techniques ranged between 79.7-86.4%, 16.7-50% and 73.9- 83.1% respectively (Table 2), while the LR presented overall sensitivity, specificity, and accuracy rates of 36.5%, 90.4% and 80.2%, respectively. The J48 was the best method in predicting patients that would relapse on the testing set, with specificity, sensitivity, and accuracy rates of 86.4%, 50% and 83.1% respectively.

### Importance of the considered features

By applying the Information gain technique (see Methods section) to the considered dataset of patients, we found that 8 of the considered features carried a significant amount of information for the correct classification of the patients’ outcome: PS, T and N stage, uTNM, Stage of the tumor, cTNM stage, tumor site, no symptoms or pain or tenesmus at diagnosis, histology and method used for the histologic definition, the presence of positive inguinal nodes, the administration of neoadjuvant CT, the treatment of an anal canal cancer relapsing after an initial surgery. In order to validate such results, we generate new predictive models using the same ML techniques, based only on the features selected by the Information gain technique. The mean accuracy was not worsened (data not shown). Interestingly, the J48 techniques improved its accuracy while considering these 8 only selected features. It means that the excluded variables have no impact on performances, or introduce only noise. This observation, confirmed by the empirical evaluation, could be of interest in understanding the actual importance of the considered features.

### Discussion (items 18-22)

We report the results of the first preliminary study exploring the potential of the innovative techniques of ML in predicting the risk of inguinal relapse in a population of 194 anal cancer patients having received or not a PII. Our results show good performances in terms of specificity, sensitivity, and accuracy of these techniques.

Subclinical inguinal metastases from anal canal cancers are not rare: their incidence is estimated at 15% to 25% in the historical surgical series [11–13].

Looking at these data, international guidelines recommend 36-45 Gy of PII in all anal cancer patients treated with radio- +/- chemotherapy [1].

Despite that, looking at the same surgical series, it should be easily argued that only 1 out 4 patients treated with PII would really benefit of the PII. These rates are lower in the early stage cancers, as it has been showed in a series by Ortholan et al., reporting a 5-year inguinal recurrence rate of 12% when omitting PII [5]. Looking at these figures, it is not strange that recent reports consider feasible and of a potential interest the reduction of the treatment fields [6], particularly in some categories of patients, presenting an increased risk of acute and late toxicity [7–9]. The overall treatment time seems to have a detrimental effect on local failure and colostomy free survival in anal cancer, and results are worst in patients presenting longer total treatment times, for example because of acute toxicity [14, 15]. On the other hand, it is also noteworthy that PII is an effective treatment: in the same study by Ortholan et al., 75 patients received PII up to a total dose of 45-50 Gy (PII group) and 106 did not receive it (no PII group) [5]. After a median followup of 61 months, 14 patients in the “no PII group” and 1 patient in the “PII group” developed an inguinal recurrence, with a 5-year cumulative rate of inguinal recurrence of 2% and 16% in “PII” and “no PII group”, respectively (p = 0.006). Finally, the real problem is to find a reliable method to deliver PII to the patients that would benefit from it, avoiding the irradiation of the 100% of the patients only to treat the 25% (or the 10%, in the case of early-stage cancers) who would really take advantage from it.

Modern highly intensity modulated radiation techniques (IMRT, Volumetric-Arc and Rotational Radiation Therapy) allow an optimal coverage of the target volumes and a better sparing of the surrounding normal tissues, with a reduction of the toxicity. In this *scenario*, the potential interest of a method allowing the further reduction of the treatment fields (and then of the toxicity) could be easily argued [16].

The results of this study indicate that ML techniques can be effectively exploited to help the radiation oncologists. Such techniques can accurately identify patients presenting a higher risk of inguinal relapse when they are not treated at the inguinal level, thus tailoring the prescription of the PII.

Another interesting aspect is that these predicting methods do not give a result in terms of probability rates (as those recently published for anal and rectal cancer [17, 18]). The output of these algorithms is a “yes/no” one (relapsing/not relapsing). The percentages are not percentages of risk of relapse, but percentages of accuracy of the algorithm.

For example, the J48 method has a confirmed accuracy of 83.1% in predicting the patients that would relapse. It means that the system classifies a very small percentage of the patients incorrectly. Moreover, AI-based methods could fit better than the classical multivariate analysis. In general it is not clear why so often AI-based methods fit better than the typical statistic approaches (i.e. logistic regression). The mathematical models behind the two families of approaches are very different and many factors, as the strong non-linearity of the problem or unusual stochastic distribution of the involved variables, could play a major role in explaining the better performances of the ML techniques. ML encompasses most of the multivariate analysis techniques. Generally speaking, most of the multivariate statistics exploit a subset of the ML approaches: usually unsupervised linear regression. In fact, ML provides a wide range of approaches that can be fruitfully exploit, as demonstrated in our work. Moreover, supervised ML techniques (as those we used) put emphasis on the prediction, i.e. the analysis is focused on identifying patterns that maximises the possibility of providing a correct prediction. On the contrary, multivariate analysis emphasises inference: patterns in the values of features are analysed regardless of their actual usefulness for predicting the outcome.

Noteworthy, Institutional treatment protocols were different in the 2 Institutions in terms of radiotherapy volumes and type of CT but, despite these important differences, ML techniques were able to correctly classify most of the patients of the testing set.

Additionally, ML techniques are able to provide some insights about the importance of considered attributes in a correct classification of the patients in the data set. The results of this analysis indicate that a smaller number of attributes are sufficient to generate good performance decision trees and so, such attributes are somehow related with the actual outcome of an anal cancer patient.

Despite the good performances of these ML-based methods, some improvements could be probably implemented in the next future, in order to increase the potential interest of these innovative approaches in the daily clinical practice.

It could be useful to integrate the variable of the timing of the relapse (i.e. to create different algorithms to predict the risk of inguinal relapse, for example, at 3 and 5 years): it could have a potential interest in deciding if irradiate or not the inguinal nodes in more elderly patients, allowing avoiding the PII in patients with shorter life expectancy.

Moreover, these results have been obtained on a monoinstitutional series (even if 62.4% of the patients have been treated in Radiotherapy Centers other than the “Leon Berard Center”): a confirmatory study performed on a large, independent population will allow to confirm and to strengthen our data.

**Figure 1:**
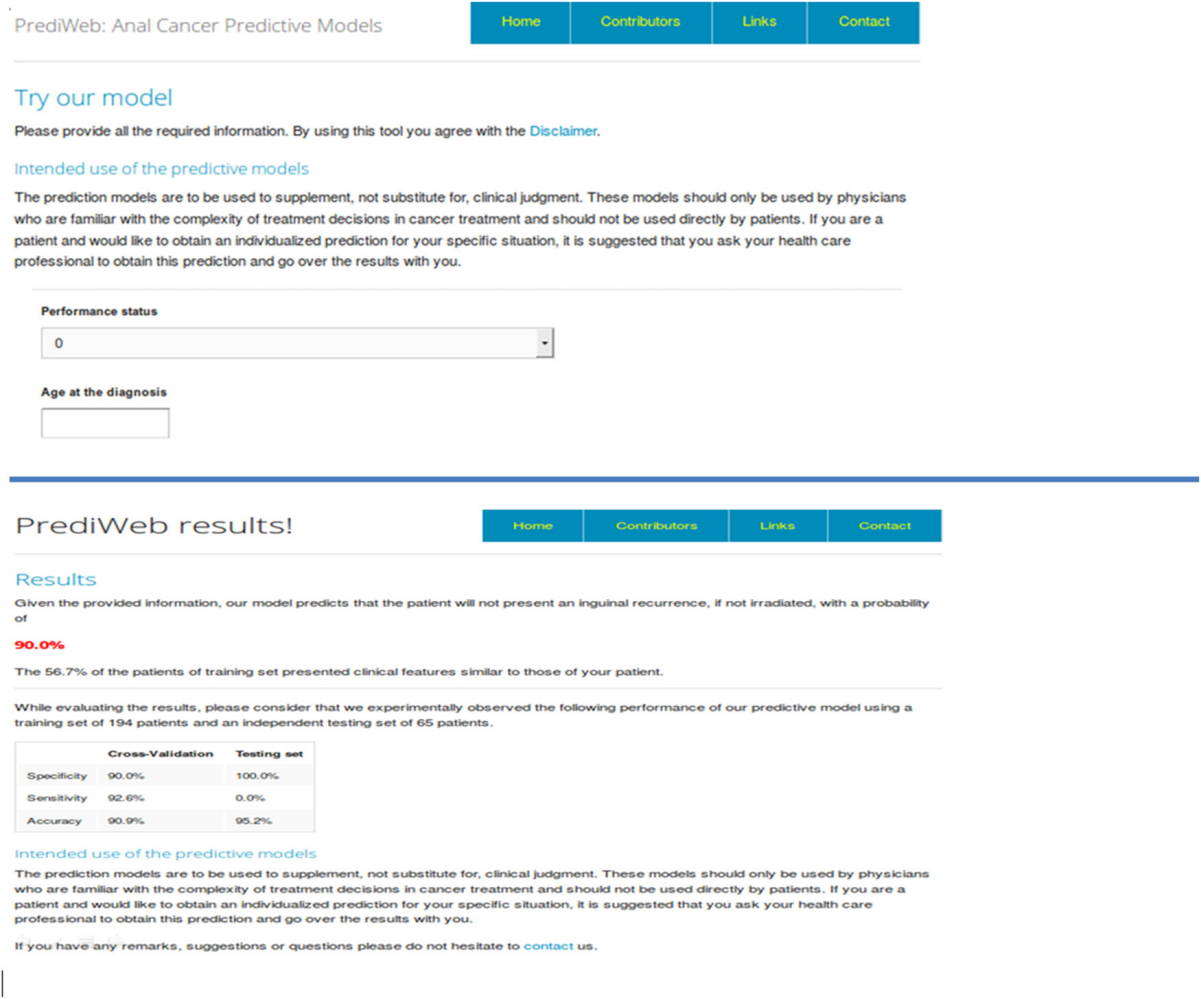
**a-b.** Screen shots taken from the PrediWeb website. The Figure 2a shows the modality of introduction of the variables (the same that have been implemented to obtain the algorithms, see Table 4), and Figure 1b shows an example of the results given by the website once all the parameters have been introduced.

These AI-based methods share the problem of needing a software (or a website) to be widely diffused: our team is already working on the creation of these informatics tools, but we prefer to confirm the performances of the algorithms in larger and/or independent population, before to finalize and diffuse them in the web. Figure 1a and 1b show some snapshots of the beta version of the open-access website that is currently under-construction and will be soon available online.

## MATERIALS AND METHODS

### The TRIPOD statement

The TRIPOD (Transparent Reporting of a multivariable prediction model for Individual Prognosis Or Diagnosis) Statement is a 22-item checklist, created to improve the reporting of studies dealing with the development and validation of diagnostic or prognostic predictive models [19]. Table 1 summarizes the items of the TRIPOD, and we followed this statement both to create and validate our model, and to present it in this article. No funds were obtained for this research (item 22).

### Participants (items 4a – 5c, 8,9 and 12)

Patients presenting an histologically proven ACC were the target population. The training set was constituted by patients consecutively addressed to the Radiation Oncology Department of the “Léon Bérard” Anticancer Center (Lyon, France) to receive brachytherapy after a first course of radiotherapy +/- chemotherapy (received in the same Center or in other Radiotherapy departments of the Rhone-Alpes Region) from may 1992 to december 2009.

The initial whole population accounted for a total of a population of 209 patients. Fifteen patients were excluded from the final analysis as at least one of the considered clinical and/or therapeutic variables was lacking (see “Experimental design” section for the variables). Finally, 194 patients were used as training sets. The Male/Female ratio was 32/162 and the median age was 64 years (range: 36-88). Median follow-up was 72.8 months.

We used an independent testing set of 65 patients affected by an histologically proven ACC consecutively treated with curative radiotherapy +/- chemotherapy at the Academic Radiation Oncology Department of the Catholic University (Rome, Italy) from Mars 1990 to August 2013. The mean follow-up was 43 months. None of the patients treated in this Department in the considered period has been excluded.

Table 3 summarizes features and differences of patients considered in the training and in the testing set, as well as their tumors characteristics (staged according to the 2002 International Union against Cancer Classification (UICC 2002) [20]) and treatment features.

**Table 3:** Description of the clinical and therapeutic features of the populations

	Testing set		Testing set	
	n.	%	n.	%
**Patients** (n)	194	100	65	100
**Sex** (n) Men Women	32 162	17 83	13 52	20 80
**Age** (y) Mean Median [Range]	63.6 64 61[36–88]	-	60.2 60 [29–88]	-
**Performance Status** 0 1 2	40 154 0	21 79 0	51 12 2	78 18 4
**SYMPTOMS** None Rectal Bleeding Pain Tumefaction/Haemorrhoids Inguinal nodes Rectal Syndrome Troubles of faecal transit Other	6 111 63 47 8 27 24 11	3 57 32 24 4 14 12 6	0 37 20 18 4 30 6 7	57 31 28 6 30 9 11
**Endorectal Echography**	130	67	21	33
**MRI**	44	23	35	54
**Description of the disease**				
**Location** (n) Anal Canal Anal canal reaching anal margin Recto-anal Anal margin Anal canal, Anal margin and Rectum	98 37 55 2 2	51 19 28 1 1	27 10 25 0 3	42 15 38 0 5
**Tumor** T1 T2 T3 T4	21 90 78 5	11 46 40 3	6 25 20 9	10 42 33 15
**Histologic Subtype** (n) Carcinoma *in situ* Large cells keratinizing squamous cell carcinoma Non keratinizing squamous cell carcinoma Basaloid squamous cell carcinoma Adenocarcinoma of rectal - anal glands type Carcinoma with small cells Undifferentiated carcinoma Other tumors (sarcomas - lymphomas - melanomas) Cloacogenic	4 53 109 15 5 1 1 4 2	2 27 56 8 3 0.5 0.5 2 1	0 29 11 10 4 1 7 1 2	44 17 15 6 1 11 1 3
**Nodal Status** N 0 N 1 N 2 N 3	140 33 14 7	72 17 7 4	31 14 13 6	48 22 20 10
**Staging TNM** I II IIIa IIIb	19 117 35 23	10 60 18 12	6 21 16 22	10 32 24 34
**Histological Procedures** Biospsy only Surgical margin R0 Surgical margin R1 Surgical margin R2	182 3 4 5	94 1 2 3	53 3 6 3	81 4.5 10 4.5
Squamous Cell Carcinoma Antigen* median value (ng/ml) range	2 0 - 11.7		5.6 0-39	
* *Available for only 15 patients in the training set*.				
*EBRT details*				
**Total Dose (Gy)** Median [range] Median Dose/fraction [range]	45Gy [36–56] 1.8Gy [1.8-3]		55 [30.6-58.5] 1.8 [1.8-2.7]	
**Pelvic Volume (patients)** “Small pelvis” (upper border up to S3) “Large pelvis” (upper border up to L5) Non available	152 39 3	78 20 2	12 53 0	18 82 0
**Inguinal irradiation (patients)** No Unilateral Bilateral	151 3 40	78 2 20	4 0 61	6 0 94
**Type of beams (patients)** Photons Photons + Perineal field (electrons) ^60^Cobalt	187 3 4	96 2 2	65 0 0	100 0 0
**Field Balistic (patients)** Orthogonal fields (2 to 4 fields) Direct perineal fields + orthogonal fields (2 to 4 fields) Intensity Modulated Radiation Therapy Not available	147 36 3 8	75 19 2 4	50 0 15 0	77 0 13 0
**Median number of fractions [range]**	25 [12–30]		27 [17–34]	
**RT Duration in days [range]**	36 [15–63]		56 [22–88]	
*BRT details*				
**Interval between RT and BRT (days)** Median range	32 12-150		-	-
**BRT technique** Low Dose Rate Pulsed Dose Rate	143 51	74 26	-	-
**Median dose [range]**	18 [10-31.7]		-	-
**Median duration of BRT in hours [range]**	22 [11–77]		-	-
**Number of sources** [range]	6 [4–12]		-	-
**Median length of sources** (cm, range)	5 [4–9]		-	-
**Median total dose RT + BRT** (Gy, range)	64 [54-76.7]		-	-
*Concomitant Chemotherapy details*				
**Schedule (patients)** No concomitant CT During the 1^st^ week of RT During the 1^st^ and 5^th^ week of RT Weekly Any other	52 18 117 7 0	27 9 60 4 0	5 5 32 7 16	8 8 49 11 24
**CT Protocol** 5FU-CDDP 5FU-MMC Weekly CDDP 40 mg 5FU-Carboplatine Weekly CDDP 30 mg 5FU-Leucovorine Xeloda -MMC Weekly MMC Tomudex-Oxaliplatin Capecitabine-Oxaliplatin	102 27 6 3 1 1 1 1 0 0	72 19 3 2 1 1 1 1 0 0	5 34 0 1 0 0 10 0 9 6	8 52 0 2 0 0 15 0 14 9
*Neoadjuvant CT*	18	9	2	3

### Outcomes (items 6a-6b)

Aim of this study was to develop a model instructed to recognize patients who would relapse if not irradiated on the inguinal groin. Because of the intrinsic nature of an automated method, we do not use any action to blind assessment of the outcome to be predicted.

### Predictors (items 7a-7b)

The performances of a classic Logistic Regression (LR) has been tested and compared to the results of the ML algorithms for the considered outcome.

For each patient, a large set of clinical or therapeutic features considered as potential predictors of microscopic inguinal involvement were included in the generation of the predictive models (see Table 4). Because of the intrinsic nature of an automated method, we do not use any action to blind assessment of the outcome to be predicted.

**Table 4:** Features considered for the development of the predictive model

Variable	Accepted values
Performance Status	From 0 to 4
Age at the diagnosis	≥ 18 years
Initial level of SCC antigen	All values ≥ 0.1
RT+/-CT after a not-curative surgical resection	Yes/No
Histologic type	*In situ* carcinoma, large cells keratinizing SCC, not keratinizing SCC, basaloid, ADK, ADK developed on a ano-rectal fistula, small cell carcinoma, undifferentiated, cloacogenic, others
Symptoms at the moment of the diagnosis	No symptoms, rectal bleeding, anal/rectal pain, anal swelling/hemorrhoids, positive inguinal nodes, rectal syndrome, defecation troubles, other.
Method used for the histological definition	Only biopsy, R0 surgical excision, R1 surgical excision, R2 surgical excision.
T side	Anal canal, anorectal junction, anal margin, anal canal with rectal extension
T stage	From 1 to 4
N stage	From 0 to 3
Staging methods	Only clinics, echoendoscopy, MRI
uTNM stage	Depending on the T and N stage established with echography
cTNM stage	Depending on the T and N stage established with clinical examination and staging examens
Neoadjuvant CT	Yes/No
Concomitant CT	Yes/No
Type of concomitant CT	5FU-CDDP 5FU-MMC Weekly CDDP 40 mg Weekly CDDP 30 mg 5FU-Carboplatine 5FU-Leucovorine Xeloda –MMC Weekly MMC Tomudex-Oxaliplatin Capecitabine-Oxaliplatin
type of inguinal irradiation	curative/prophylactic

Legend: SCC = squamous cell carcinoma; ADK = adenocarcinoma; CDDP = cisplatin; 5-FU = 5-fluorouracile; MMC = mitomycine.

### Statistical methods (items 10a-e and 11)

The correct selection of the best classification technique is crucial: it should be an automated system being reliable, allowing accurate predictions and, at the same time, easy to be explained and represented. We decided to adopt ML techniques based on the decision trees (the J48 [21], the Random Tree [22] and the Random Forest [23]). The methodology that we adopted has been previously described and detailed in a previous study by our group [24].

Two risk groups were created, according to the outcome of the treatment in terms of relapse. The largest one accounted for 160 patients, and it referred to patients who, regardless to the received treatment, did not relapse. The risk group that the model should predict was the alternative one, including only the relapsing patients. This class accounted for 34 patients. As it could be easily argued, these population are quite unbalanced. Thus, we adopted the random oversampling to take into account the imbalanced patients distribution among these 2 groups [25]. Random oversampling is frequently adopted in AI studies, and it increases the number of elements of the less represented class (relapsing patients, in our population) by randomly considering more than once some of these patients. After the application of this technique, classes had a distribution of about 70%-30% of, respectively, non-relapsing and relapsing patients [26]. Finally, the considered population is composed by 230 patients; 160 of them did not relapse, while 70 of them are members of the relapsing class. It is worth to note that oversampling could lead to overfitting, which results in over-structured models that are too focused on training population.

As it is also stated in the TRIPOD statement, it should be avoided to evaluate the performances of a model on the same data from which the model was developed. Indeed, it could overestimate its performances, owing to overfitting (too few outcome events relative to the number of candidate predictors**).** For this reason, some forms of internal validation, as bootstrapping or cross-validation, should always be part of the development of a new prediction model. This is also clearly stated in the TRIPOD indications. In our study, each of the selected classifying techniques was trained on the previously described data sets separately, and the resulting predictive models were then evaluated using a k-fold cross-validation strategy [27]. Models have been evaluated by considering their accuracy, specificity and sensitivity. Accuracy indicates the proportion of patients of the given class correctly classified. Sensitivity is the ability of the model to correctly classify a patient in a given class. Specificity relates to the ability of the generated algorithm to identify and classify patients as not to be members of a given class, and that are actually not members of the considered class. In order to compare the performances of ML techniques with a more common statistical approach, we trained a LR model using the same k-fold cross-validation schema. The used algorithm was the one implemented in R software (version 3.0.0) [28] ant it was based on the generalized linear model inspired from Hastie et al [29].

Finally, performances of the obtained models have been verified on a testing set of 65 anal canal cancer patients treated in another Radiotherapy Department (Catholic University, Rome, Italy).

Last but not least, we decided to define an intuitive ordering of the importance of the considered features, in order to assess those having a major role in the prediction. We applied the Information gain technique [30, 31]. This technique, widely adopted in ML applications, is based on an evaluation of the information that each feature carries with regard to the class to predict. Globally, it measures the information that is lost when a single feature, or a subset of the available features, is used to approximate the class to predict.

## CONCLUSION

ML-based methods seem promising tools in predicting patients who are the best candidates to the PII, with very good performances in terms of sensibility, sensitivity and accuracy. ML could potentially help the Radiation Oncologist in the selection not only of those patients who would benefit of the PII, but also of those that would only be exposed to the potential toxicity of this treatment, increasing the therapeutic ratio of the treatments. These interesting results should to be confirmed in larger and/or independent populations of ACC patients.
